# Nanoscale Growth Initiation as a Pathway to Improve the Earth-Abundant Absorber Zinc Phosphide

**DOI:** 10.1021/acsaem.1c02484

**Published:** 2021-10-04

**Authors:** Simon Escobar Steinvall, Elias Z. Stutz, Rajrupa Paul, Mahdi Zamani, Jean-Baptiste Leran, Mirjana Dimitrievska, Anna Fontcuberta i Morral

**Affiliations:** † Laboratory of Semiconductor Materials, Institute of Materials, Ecole Polytechnique Fédérale de Lausanne, 1015 Lausanne, Switzerland; ‡ Center for Analysis and Synthesis and NanoLund, Lund University, Box 124, 221 00 Lund, Sweden; § Institute of Physics, Ecole Polytechnique Fédérale de Lausanne, 1015 Lausanne, Switzerland

**Keywords:** zinc phosphide, earth-abundant, absorber, nanoscale growth, vapor−liquid−solid, selective area epitaxy

## Abstract

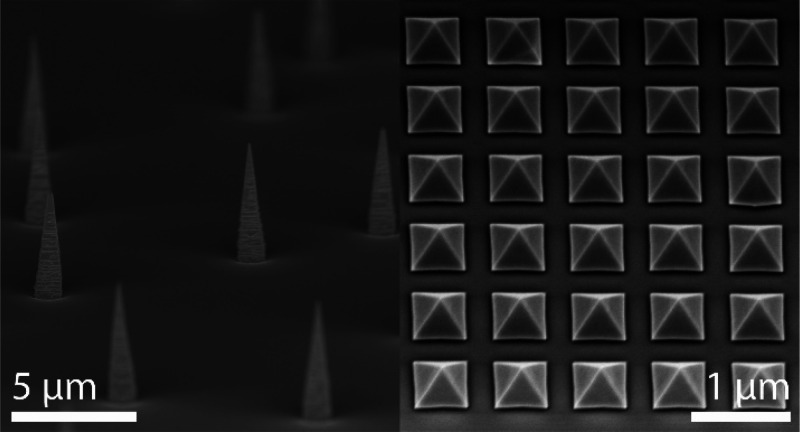

Growth approaches that limit the interface area between layers to nanoscale regions are emerging as a promising pathway to limit the interface defect formation due to mismatching lattice parameters or thermal expansion coefficient. Interfacial defect mitigation is of great interest in photovoltaics as it opens up more material combinations for use in devices. Herein, an overview of the vapor–liquid–solid and selective area epitaxy growth approaches applied to zinc phosphide (Zn_3_P_2_), an earth-abundant absorber material, is presented. First, we show how different morphologies, including nanowires, nanopyramids, and thin films, can be achieved by tuning the growth conditions and growth mechanisms. The growth conditions are also shown to greatly impact the defect structure and composition of the grown material, which can vary considerably from the ideal stoichiometry (Zn_3_P_2_). Finally, the functional properties are characterized. The direct band gap could accurately be determined at 1.50 ± 0.1 eV, and through complementary density functional theory calculations, we can identify a range of higher-order band gap transitions observed through valence electron energy loss spectroscopy and cathodoluminescence. Furthermore, we outline the formation of rotated domains inside of the material, which are a potential origin of defect transitions that have been long observed in zinc phosphide but not yet explained. The basic understanding provided reinvigorates the potential use of earth-abundant II–V semiconductors in photovoltaic technology. Moreover, the transferrable nanoscale growth approaches have the potential to be applied to other material systems, as they mitigate the constraints of substrate–material combinations causing interface defects.

## Introduction

Performance in photovoltaic (PV) devices is often crippled by the presence of defects, to an extent by bulk defects, but most often interface defects are the limiting factor.^1−4^ This may include dangling bonds due to incoherent interfaces, which facilitate charge recombination.^1−4^ In addition, PV devices require several layers in order to effectively absorb the incoming light, to separate photogenerated electron–hole pairs, and finally to extract the charge carriers to perform work. All layers would ideally be grown epitaxially to create pristine interfaces. Unfortunately, the many layers required are likely to have a range of different lattice parameters, which significantly complicates this process for most material combinations. In the past couple of decades, nanotechnology approaches have been developed to mitigate the influence of lattice mismatch in heterostructure formation.

One area in particular that has explored defect-free heterostructure formation is the field of compound semiconductor nanowires.^5,6^ By reducing two of the dimensions to nanometer lengths, one reduces the amount of strain energy build up at the interface due to lattice mismatch while also activating additional elastic radial stress relaxation mechanisms.^7−9^ Although various material systems have been explored in the form of nanowires, the bulk of the research is based on III–V nanowires grown by the vapor–liquid–solid (VLS) method.^10,11^ III–V nanowires have been of particular interest for photovoltaic applications and III–V integration on silicon for CMOS compatible optoelectronic applications.^12−15^ With regard to PV applications, nanowires have been of particular interest as their nanophotonic properties, which allow for a reduction in the usage of critical raw materials, as a nanowire array can absorb as much light as a thin film due to their enhanced absorption cross-section.^16,17^ Furthermore, the directivity of anisotropic nanostructures makes it possible for them to surpass the maximum theoretical efficiency as dictated by the thermodynamic balance limit, albeit the highest conversion efficiency demonstrated to date by bottom-up grown nanowire devices is 15.3% (17.8% for top-down).^12,13,17,18^

**Figure 1:**
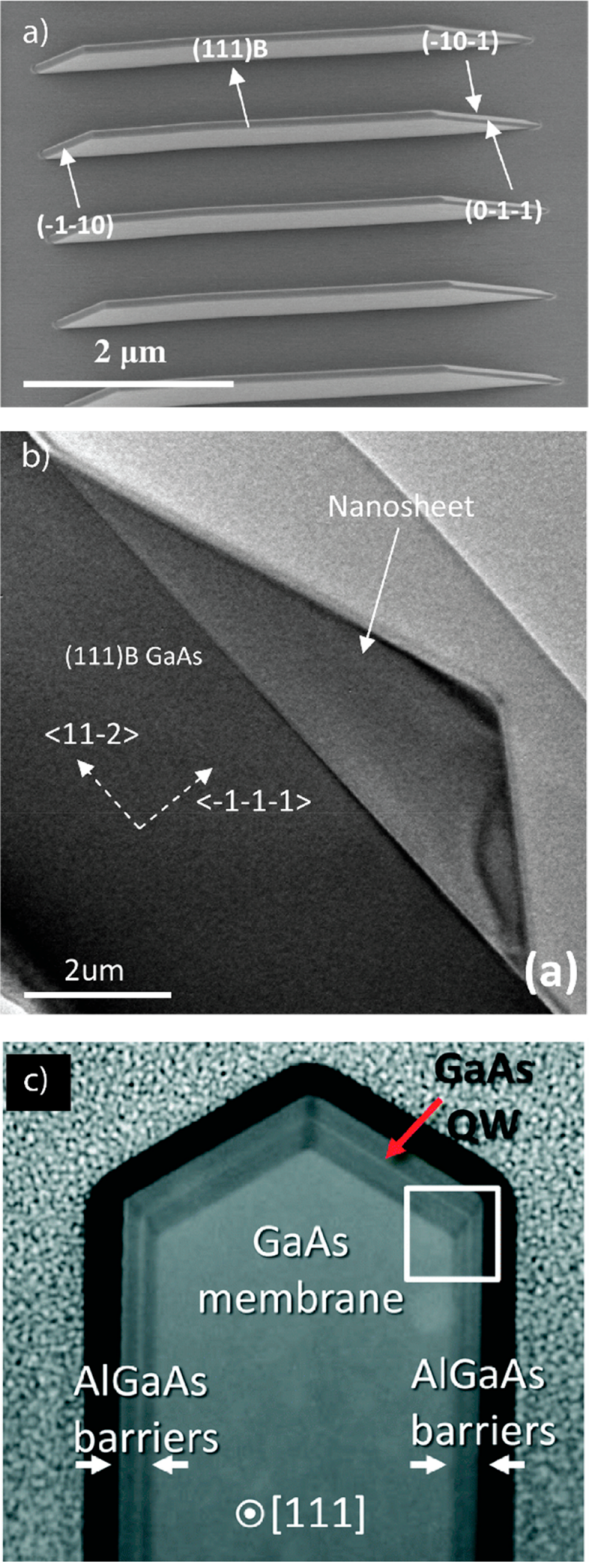
(a) SEM image of GaAs nanomembranes. Reprinted with permission from ref (20). Copyright 2013 American Chemical Society. (b) HR-TEM image of a GaAs nanomembranes showing no defects. Reprinted with permission from ref (20). Copyright 2013 American Chemical Society. (c) Cross-sectional HAADF-STEM image of a GaAs-AlGaAs heterostructure grown on a nanomembrane. Reprinted with permission from ref (21). Copyright 2015 Royal Society of Chemistry.

Recently, interest has sparked for synthesis methods that control the morphology of the vapor–solid grown structure through a patterned mask that limits the interface area, so-called selective area epitaxy/growth (SAE/SAG).^19^ An advantage of this method is that no catalyst is used (as compared to VLS growth). Differently from VLS, this method can be used to grow defect-free horizontal nanowires/structures as well as heterostructures with pristine interfaces as showcased in Figure 1.^20,21^ Since then, this approach has been increasingly researched for nanowire networks for electronic and quantum computing applications.^22−25^ The technique currently used to define the patterns at research level is mainly electron beam lithography, which exhibits limited potential scalability due to its low throughput. However, once the pattern has been optimized the process is in theory transferable to more scalable processes such as stepper or nanoimprint lithography. Nonetheless, the promise of high-quality interfaces and crystals are also desirable for photovoltaic applications.

One material in particular that stands to gain from these potentially defect-free synthesis routes is zinc phosphide (Zn_3_P_2_). Zinc phosphide is an earth-abundant absorber material with a direct band gap close to the optimum for PV applications (1.50 eV),^26^ a high optical absorption in the solar spectrum (10^–4^–10^–5^ cm^–1^),^27,28^ and a long minority carrier diffusion length (∼10 μm),^26^ which combined with its stability in ambient conditions^29^ make it an ideal material for low-cost and large-scale thin film PV applications.^30^ The high absorption enables ultrathin and lightweight PV devices, which are of interest for flexible optoelectronics and the Internet of Things, among other potential applications. Despite its attractive properties there has been limited success in developing this material and the record device (∼6% conversion efficiency) was reported 40 years ago.^30^ The factors that have limited this material are its large tetragonal unit cell (*a* = *b* = 8.08 Å; *c* = 11.39 Å)^31,32^ and coefficient of thermal expansion.^33^ This makes epitaxial synthesis on commercially available substrates rather difficult, due to either lattice mismatch or strain build-up when cooling down postsynthesis. Furthermore, its complex defect chemistry additionally complicates the efficient charge extraction through either homojunction or heterojunction formation.^34−37^ To overcome these challenges we have investigated the epitaxial growth of zinc phosphide nanostructures, which we will explore in more detail next.

## Our Work

Our work provides fundamental studies on the epitaxial growth of zinc phosphide by molecular beam epitaxy. This technique allows for low-growth temperatures and precise control of growth-related parameters. As growth substrates we have used InP and graphene. InP diverges from the earth-abundant aim of the research and would need to be replaced for final applications. However, as an initial step to demonstrate high-quality material, it does offer an ideal platform for fundamental studies. Its unit cell (*a* = 5.83 Å)^38^ is relatively close to that of the pseudocubic reconstruction of zinc phosphide (*a* = 5.73 Å), which is achieved by disregarding the systematic absences of the zinc atoms in its pseudofluoritic lattice.^39^ While GaAs offers a closer match (*a* = 5.65 Å), it is not ideal due to the formation of a GaP interfacial layer, which may affect the final performance.^40^ Graphene as a substrate is of interest for van der Waals epitaxy where there are no covalent bonds forming at the interface, and thus completely ignores any lattice mismatch.^41^ With this in mind, we now move on to how the growth was achieved.

## Growth and Crystalline Properties

**Figure 2:**
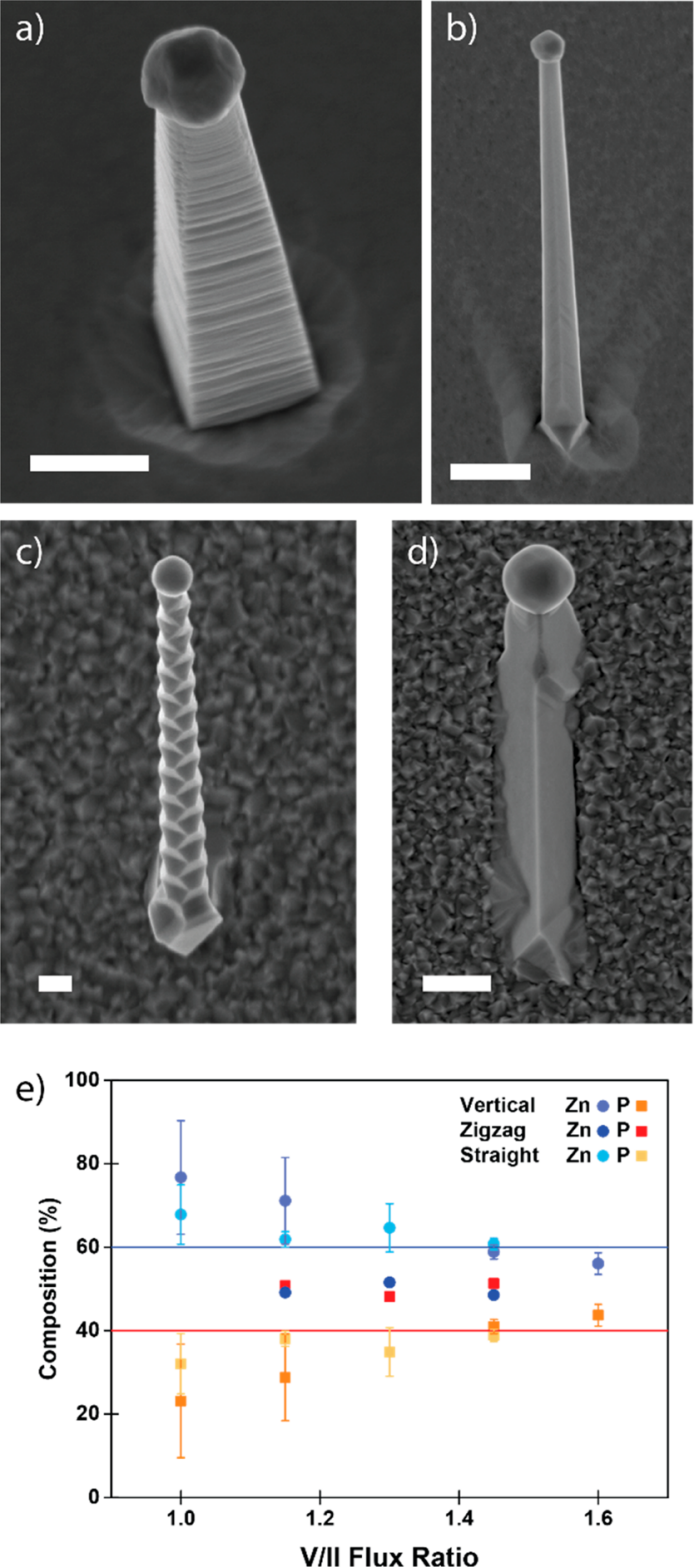
Representative SEM images of (a) vertical (tilted view), (b) straight-tilted, (c) zigzag, and (d) crawling zinc phosphide nanowires (scale bars, 500 nm). (e) Graph showing the nanowire composition as a function of growth conditions and morphology. Reprinted with permission from ref (49). Copyright 2020 Royal Society of Chemistry.

VLS growth relies on the selective absorption of the precursors into a liquid catalyst particle, which on supersaturation will selectively precipitate in one direction to create a 1D structure.^42^ The different catalysts explored for zinc phosphide include gold, bismuth, tin, and indium, the latter which will be the focus here.^39,43−49^ Indium nanoparticles were generated by reacting the InP substrate in ultrahigh-vacuum conditions with zinc through a 5 min predeposition step. We were able to observe four distinct growth morphologies when phosphorus and zinc were supplied simultaneously as vapor-phase precursors. The morphology was a function of the growth temperature and the phosphorus to zinc flux ratio (V/II ratio), as shown in Figure 2a–d. Out of the different morphologies, the vertical one is of greatest interest for PV applications as it is in the ideal configuration to exploit its nanophotonic properties. Transmission electron microscopy (TEM) and Raman spectroscopy were used to determine the nanowires’ growth direction. Vertical nanowires (Figure 2a) grew along the *c*-axis of zinc phosphide, making the epitaxial relationship [001]_Zn_3_P_2__/[001]_InP_.^49,50^ The zigzag and straight-tilted nanowires (Figure 2b,c) grew perpendicular to (101) planes instead.^49^ In the pseudocubic reconstruction this equates to a [111] growth direction, which is the most commonly observed one for zincblende nanowires of other materials.^10,11^ The difference in morphology/faceting seems to be related to growth temperature, where zigzag nanowires form at higher temperatures.^49^

Absolute morphology engineering turned out to be challenging. Preliminary results indicate that it relates to the catalyst formation mechanism utilized. Because the substrate is consumed to form the catalyst, one cannot accurately control the exact plane at the interface, which is key to defining the kind of structure that will ultimately form. Similar to what was proposed for InP nanowires, when the (001) plane is exposed, we achieve vertical growth, while (111) planes result in the growth of alternative growth directions.^51^

Next, we delved deeper into the characterization of the defects in zinc phosphide nanowires. The vertical nanowires were studied using aberration-corrected high-angle annular dark-field scanning transmission electron microscopy (AC HAADF-STEM). No stacking faults could be observed along the growth direction.^49^ However, we observed the formation of nanoclusters of InP on the surface, indicating that the catalyst particle was consumed during growth to yield a tapered shape, albeit radial vapor–solid growth to a certain extent cannot be discounted.^49^ Compositional analysis was also performed of the vertical nanowires using energy-dispersive X-ray spectroscopy (EDX). Unexpectedly, we observed that the composition could deviate greatly from the ideal stoichiometry (Zn_3_P_2_) into both zinc-rich and phosphorus-rich regions. The final composition is a function of the V/II ratio used during synthesis as shown in Figure 2e.^49^ While defects could not be directly observed here, defect-related transitions were observed through optoelectronic characterization as will be explained in more detail in the next section.

**Figure 3:**
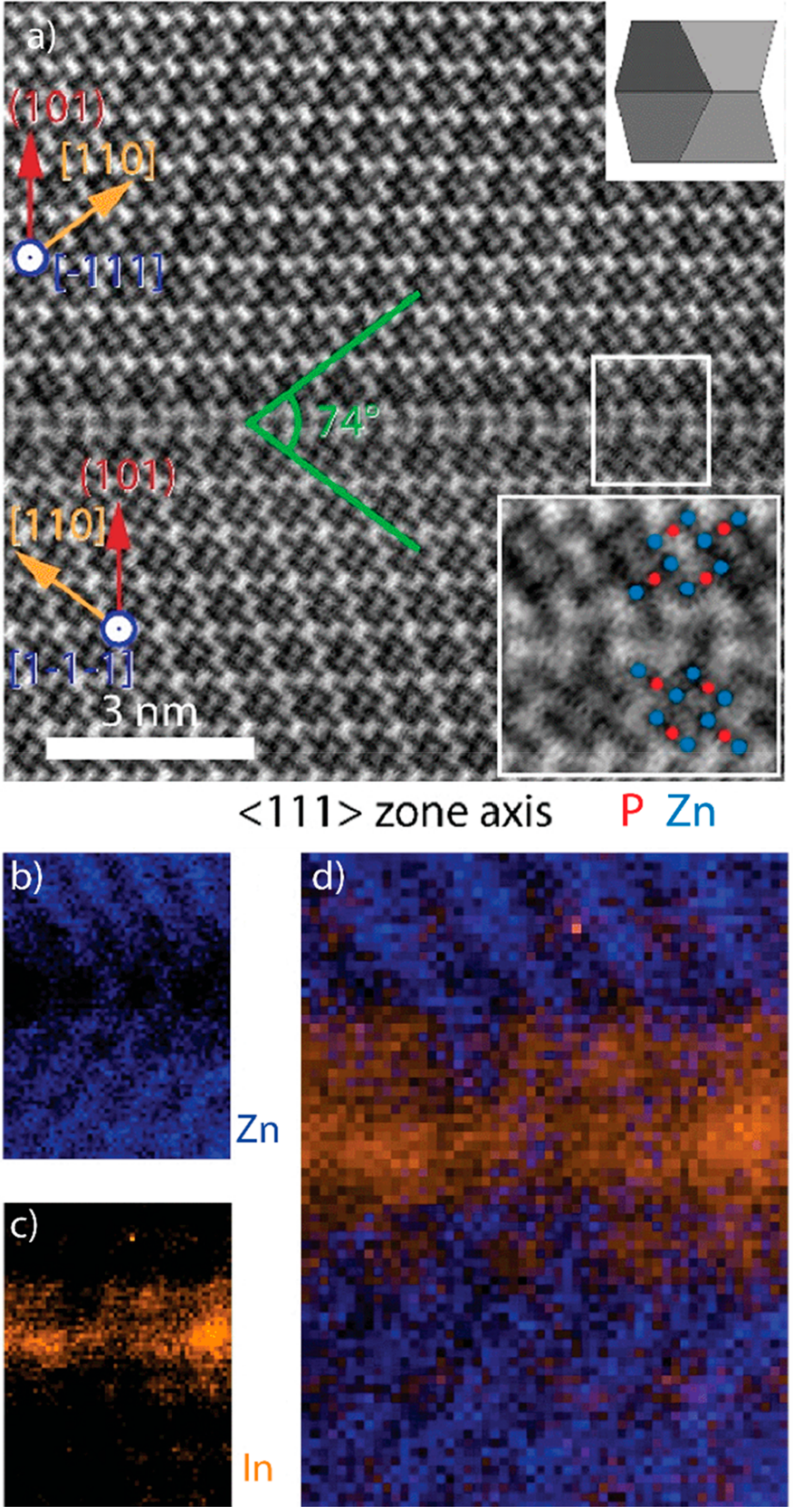
(a) AC-BF-STEM image of the heterotwin region with inset showing the atomic positions. Core-loss EELS maps of (b) Zn, (c) In, and the combined image (d). Reprinted with permission from ref (54). Copyright 2020 Royal Society of Chemistry.

The tilted nanowire morphologies showed different defect behavior compared to the vertical nanowires. The straight-tilted nanowires were shown to contain multiple stacking faults perpendicular to the (001) planes in the wire.^49^ The zigzag nanowires on the other hand showed quite different types of defects. The zigzag structure is achieved through a so-called twin plane superlattice.^52,53^ Here, each section is rotated with respect to each other, which is facilitated by a heterotwin. The oscillatory nature of the superlattice is a consequence of the catalysts’ stability on the top facet, which has a cross-section that goes from hexagonal to truncated triangular.^54^ When the cross-section becomes more triangular in nature, the normalized surface energy increases. Lowering of the system energy is achieved by the formation of a (hetero)twin that causes the cross-section to return toward a more stable hexagon.^54^ However, zinc phosphide has a tetragonal structure that does not readily form standard twins. Upon closer inspection by AC bright-field (BF) and HAADF-STEM in combination with atomic resolution core-loss electron energy loss spectroscopy (EELS) mapping, we observed that the mirror plane is an indium-rich discontinuity, forming a heterotwin as shown in Figure 3. The inclusion of indium with a higher valency facilitates the rotation of the crystal sections.^54^

**Figure 4:**
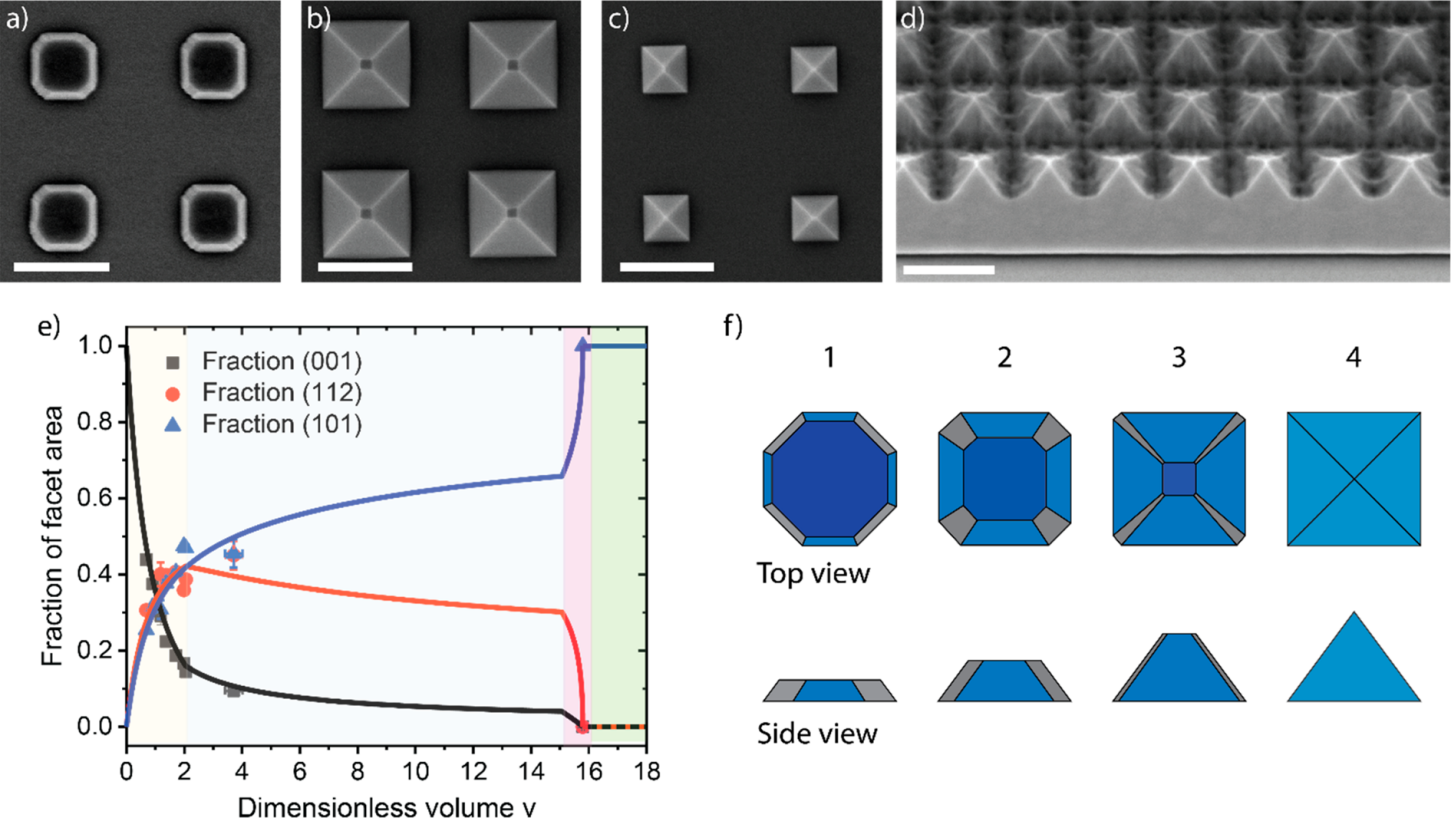
(a–d) Representative SEM images of SAE-grown nanopyramids at varying degrees of completion and coalescence (scale bars, 500 nm). (e) Graph showing the fraction of the different facets as a function of the dimensionless volume. (f) Illustrations of the nanostructure shape for the different regions in panel e. (a–c, e, f) Reprinted with permission from ref (57). Copyright 2021 American Chemical Society.

The other approach utilized for the successful growth of zinc phosphide nanostructures is SAE, where growth is achieved in selectively etched holes in a mask layer.^10,19^ For zinc phosphide we used a combination of a SiO_2_ mask on a InP (100) substrate.^55^ The morphology of the nanostructure is a function of the shape of the hole. Nanopyramids are the result for circular holes, while nanowires, or even nanowire networks, are achieved from elongated slits as shown in Figure 4.^55−57^ However, these shapes are only observed after a couple of intermediate stages. The intermediate morphology as zinc phosphide outgrows the hole depends on the relative surface energies of the dominating facets, namely, (001), (112), and (101). DFT calculations showed that *E*_(101)_ < *E*_(112)_ < *E*_(001)_.^55,58,59^ The morphology evolution can be determined through a dimensionless geometric parameter based on the relative surface energies and initial hole size.^57^ The facet fractions and shape evolution based on this model are shown in Figure 4. The inherent pyramidal morphology is ideal for light trapping in the zinc phosphide layer, which is advantageous for PV applications.^60−62^

The interface between the indium phosphide substrate and zinc phosphide was investigated using HR-TEM and AC HAADF-STEM of FIB lamellae and did not include any misfit dislocations.^55,56^ The interface roughness observed was due to non-optimized pattern etching. The epitaxial relationship observed was [001]_Zn_3_P_2__/[001]_InP_, making the vertical growth direction of zinc phosphide the *c*-axis ([001]_Zn_3_P_2__). This combination results in the minimum strain in the zinc phosphide.^56^ Most of the epitaxial strain is relaxed by the time the structure starts to grow out of the hole in the mask. One peculiar defect we did observe was the presence of rotated domains. These domains are related to when the growth front shifts from a (001)_Zn_3_P_2__ to a (101)_Zn_3_P_2__ surface and is characterized by a 120° rotation around the (101)_Zn_3_P_2__ plane.^55,56^ The rotated defects were mainly observed in the edges when the zinc phosphide starts to overgrow the mask. However, they could also be observed within the nanowires when {101}_Zn_3_P_2__ surfaces randomly form during early stages of the growth and provide nucleation sites for these domains.^56^ As they continued to grow, they also form extended (100)_Zn_3_P_2__//(112)_Zn_3_P_2__ interfaces. Interestingly, DFT calculations of the formed rotated interfaces did not observe the formation of any dangling bonds or mid-band-gap states at the (101)_Zn_3_P_2__ rotated interface, albeit a slight local reduction in the band gap was observed, which may be a potential source of sub-band-gap recombination. The (100)_Zn_3_P_2__//(112)_Zn_3_P_2__ interface combination was unfortunately not as ideal, and shallow acceptor states were observed (discussed in more detail further down).^56^

If the nanopyramids or nanowires are allowed to grow laterally for a sufficient amount of time (depending on the initial pitch and hole size), they eventually coalesce and form thin films.^55^ In theory it is possible to tune the pitch to avoid the formation of grain boundaries. Still, because the grain boundaries are not traversing the carrier extraction path, their potential influence should be minimal. The formation of Zn_3_P_2_ thin films through coalescence of nanoflakes was also observed through van der Waals epitaxy on graphene.^41^ So far, this approach did not lead to a perfect alignment between all neighboring nuclei due to the weak substrate interactions.

## Functional Properties

To determine the applicability and direction of improvement of the structure, we then investigated the functional properties of the zinc phosphide nanostructures grown by both VLS and SAE. Studies of VLS-grown nanowires using valence electron energy loss spectroscopy (VEELS) combined with DFT,^63^ cathodoluminescence (CL),^49,54^ and absorptance measurements^28^ have been performed to evaluate their optoelectronic properties. On the other hand, SAE-grown zinc phosphide nanostructures were evaluated through photoluminescence (PL),^55^ conductive atomic force microscopy (c-AFM),^55^ and DFT calculations.^56^

**Figure 5:**
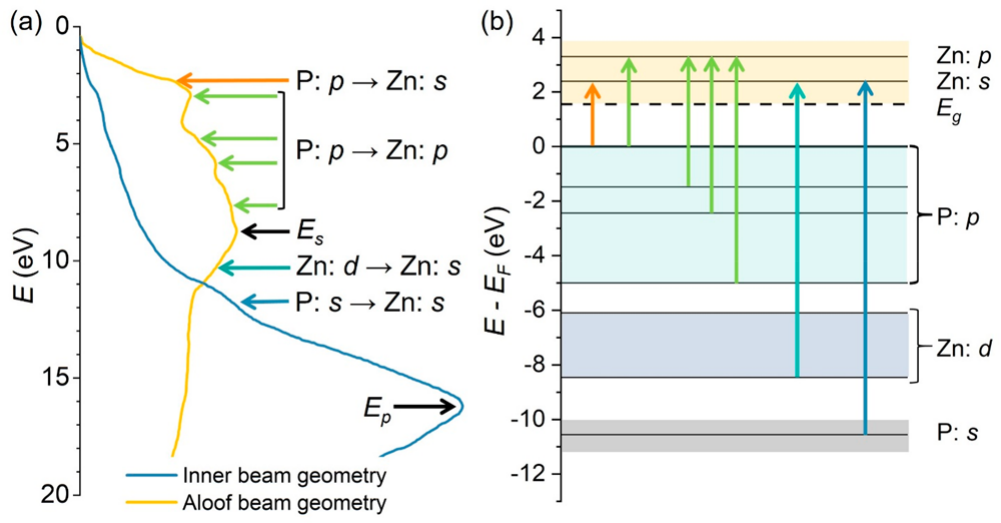
(a) Identification of interband transitions in VEELS spectra based on the band structure and P(DOS) obtained from DFT. The arrows indicate the interband transitions, while *E*_s_ and *E*_p_ denote the energies of the surface and the volume plasmons. (b) Schematic energy level diagram for Zn_3_P_2_. Energies are taken from the band structure and DOS calculations and are calibrated with respect to energy zero at the top of the valence band. Reprinted with permission from ref (63). Copyright 2021 The Authors, under Creative Commons Attribution 4.0 International license, published by Wiley-VCH.

AC and monochromated VEELS were performed on a VLS-grown zigzag nanowire. Recent technological advances improving spatial and spectral resolution have made this technique an emerging powerful tool to analyze the full electronic structure and plasmonic properties of materials. The measured VEEL spectrum, shown in Figure 5, contains a plethora of information which we could match with theoretical predictions from DFT.^63^ We could therefore determine the energy of zinc phosphide’s fundamental direct band gap (1.50 ± 0.10 eV) and also determine all higher-order electron transitions and plasmonic contributions.^63^

Using CL we further investigated the tunability of the functional properties as functions of composition and morphology.^49^ Cryo-CL measurements (at 10 K) comparing vertical and zigzag nanowires showed very different characteristics between the two morphologies. Vertical nanowires showed greater emission at the base, while lowering in intensity and a slight red shift of the signal were observed toward the top of the nanowires. Also, the emission energy of vertical nanowires was found to be dependent on the their composition, varying from 1.35 eV (Zn-rich) to 1.31 eV (P-rich).^49^ Conversely, the zigzag nanowires showed a position-independent emission at 1.43 eV.^49^ The position independence indicates that there was no observable effect of the heterotwins on the emission characteristics. However, this might also be due to the increased diffusion length at cryogenic temperatures. To better ascertain the position dependence we repeated the measurements at room temperature to reduce the diffusion length and improve the spatial resolution.^54^ These measurements showed the emergence of band gap (1.50 eV) and above-band-gap (1.67 eV) emission from a higher-order conduction band transition, but we were still unable to observe any influence of the heterotwins.^54,63,64^

Single nanowire optical absorption measurements were also performed on the zigzag nanowires in the 1.96–2.54 eV range using a suspended nanothermometer.^28^ The zigzag nanowire showed a 5-fold greater optical absorptance when compared to gallium arsenide nanowires in this energy range. This is explained through the large number of higher-order conduction bands creating a high density of states for above-band-gap energy transitions, increasing the absorptance and making it an ideal material for PV applications.^63,64^ Further studies to discern the influence of stoichiometry and morphology on these properties are currently underway.

**Figure 6:**
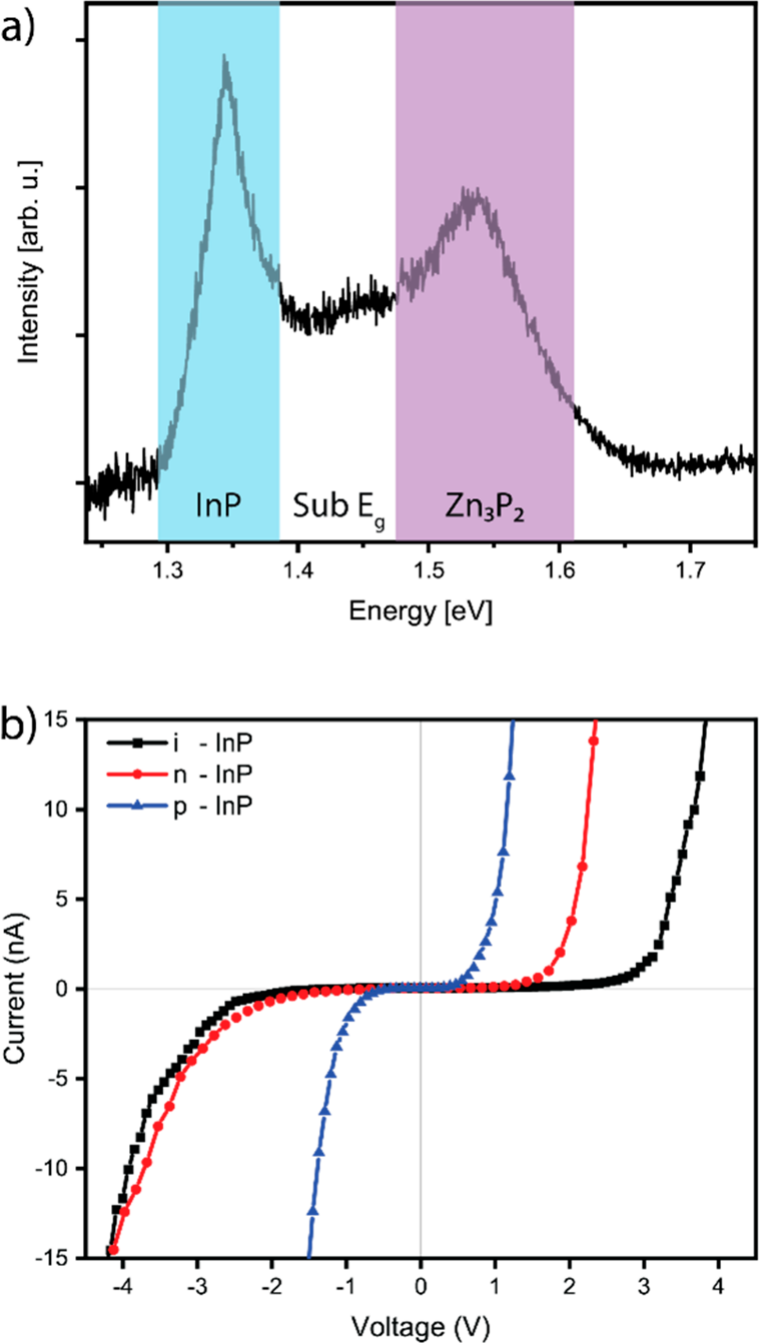
(a) PL spectrum acquired from an array with a 600 nm pitch and 150 nm nominal hole size, showing the indium phosphide, zinc phosphide, and defect emission. (b) c-AFM *I*–*V* curves of zinc phosphide grown on intrinsic, n-type, and p-type indium phosphide. Reprinted with permission from ref (55). Copyright 2021 Royal Society of Chemistry.

The optoelectronic properties of SAE-grown zinc phosphide were assessed through room-temperature PL. The PL spectrum, Figure 6a, showed a clear band gap transition at 1.53 eV with some sub-band-gap emission.^55^ The clear presence of the band gap emission is indicative of the high crystalline quality of the SAE-grown structures. A potential origin of the sub-band-gap emission may be the interfaces between the rotated domains. The previously mentioned DFT calculations show the formation of inter-band-gap states at 0.10, 0.21, and 0.38 eV above the valence band.^56^ The sub-band-gap emission observed is in this range, and these transition energies also coincide with defect energies that have been experimentally observed previously by other studies into zinc phosphide.^41,49,55,65−70^

Finally, c-AFM was used to determine the electrical properties of zinc phosphide nanopyramids grown on different substrates, as shown in the representative *I*–*V* curves in Figure 6b.^55^ The sample of greatest interest is the zinc phosphide grown on n-type indium phosphide. This combination shows anisotropic *I*–*V* characteristics, indicative of diode behavior stemming from p–n junction formation, while the other combinations (i and p) show more symmetric trends. Therefore, we assume that the as-grown nanopyramids are p-type. However, there is a potential influence of pitch and hole size, as it affects the composition, which is still in the process of being assessed.

## Summary and Outlook

In conclusion, VLS and SAE are promising routes for the growth of zinc phosphide nanostructures with properties suitable for earth-abundant PV applications. To fully grasp the potential of this concept, there are still aspects in need of elucidation. First, these approaches show great control over the composition of the material, but the exact impact on, among others, the electrical properties still needs further clarification in order to be fully optimized for devices. Second, the current growth utilizes substrates with a critical raw material (indium), and the process should therefore either be moved to earth-abundant substrates or use approaches which allow for recycling of the substrate. Both of these approaches are compatible with nanostructure growth, as they allow for either (i) growth at high lattice mismatch due to the small interface area and (ii) the potential exfoliation of the grown structures and transfer to other substrates for device fabrication, allowing the growth substrate to be reused. Initial trials of the first approach of SAE-grown zinc phosphide on silicon substrates look promising, but are still in the early stages. The third aspect is charge separation mechanisms, either through the exploration of compatible heterojunction materials or dopants for controlled homojunction formation. Bottom-up growth of nanostructures facilitates exploration of this aspect due to the emergence of additional stress relaxation mechanisms, facilitating homojunction formation and the possibility to homogeneously introduce dopants during the growth stage. Finally, the approaches described here to improve the material and interface quality are not limited to zinc phosphide and may be of great interest to apply to other emerging earth-abundant photovoltaic systems.
